# Assessing maternal knowledge of neonatal danger signs in Egypt: a cross-sectional study

**DOI:** 10.1038/s41598-026-50756-z

**Published:** 2026-05-08

**Authors:** Esraa Abdellatif Hammouda, Marwa Abdelwahab Hassan, Nesma Abbas Hassan, Nayera Mohamed Abdelmoez, Amal M. Shouair, Rasha Ashmawy, Saddam Abdelazim, Mariam Zaghloul Mohamed Daowd, Hala S. M. Abdelmotogaly, Shrouq Sayed Abdelrazek, Asmaa Rohym, Abdelrahman Shawky Refaee, Rania Adel El-Morsy, Ramy Mohamed Ghazy

**Affiliations:** 1 Medical Research Institute, Alexandria University, Alexandria, Egypt; 2 Clinical Research Department, El-Raml Pediatric Hospital, Ministry of Health and Population, Alexandria, Egypt; 3 Clinical Research Department, El-Anfoushy Pediatric Hospital, Ministry of Health and Population, Alexandria, Egypt; 4 Clinical Research unit, Asyut Health Directorate, Ministry of Health and Population, Asyut, Egypt; 5 Beheira Health Directorate, Ministry of Health and Population, Beheira, Egypt; 6 Clinical Research Department, Maamora Chest Hospital, Alexandria, Egypt; 7 Clinical Research Department, Minya Health Directorate, Ministry of Health and Population, Minya, Egypt; 8 Lecturer of Pediatrics, Al-Galaa Teaching Hospital, General Organization of Teaching Hospitals and Institutes (GOTHI), Cairo, Egypt; 9 Lecturer of Pediatric and Neonatology department, Faculty of Medicine, New Valley University, Kharga, Egypt; 10 Clinical Research Department, Badrashin Hospital, Ministry of Health and Population, Giza, Egypt; 11 Clinical Research Department, Fayoum Health Directorate, Ministry of Health and Population, Fayoum, Egypt; 12 Lecturer of Pediatrics, October University for Modern Sciences and Arts (MSA), Cairo, Egypt; 13 Drug information specialist, Sherbin Central Hospital, Ministry of Health and Population, Dakahlia, Egypt; 14 Tropical Health Department, High Institute of Public Health, Alexandria University, Alexandria, Egypt

**Keywords:** Health care, Medical research, Risk factors

## Abstract

Maternal knowledge regarding the neonatal danger signs (NDSs) plays a fundamental role in early detecting neonatal health conditions, decreasing untreated symptoms, and improving optimal infant development. This study aimed to determine the level of maternal knowledge about NDSs and identify associated socio-demographic factors among Egyptian mothers. A cross-sectional study was conducted in healthcare facilities through face-to-face interviews from January to March 2025. We included mothers older than 18 years with infants aged 1–24 months. Mothers were recruited through a multistage stratified sampling technique from nine Egyptian governments selected from all Egyptian geographical sectors. The Arabic Questionnaire to assess the Knowledge of Neonatal Danger Signs (AQ-KNDS) was used for assessing maternal knowledge with a score ranged from 1 to 16. Multiple linear regression model identified predictors of knowledge score. Of 1831 participating mothers representing 96.3% response rate, 31% were under 25, 97.3% were married, and 58.7% were living in urban areas. The median score of maternal knowledge was 14.0 (IQR: 13.0–16.0). 75.8% of mothers scored above 75.0%. Beheira governorate had the highest median knowledge score while New Valley had the lowest (15.0 (13.3–16.0) vs. 13.0 (12.0–14.0), *P* < 0.001). Higher-educated mothers had a significantly higher knowledge scores compared to lower education (median (IQR) 15.0 (13.0–16.0) vs. 14.0 (13.0–16.0), *P* = 0.006). The multivariable linear regression model predicted the higher knowledge score was due to living in rural areas ( β = 0.44; 95% CI 0.23–0.66); *P* = 0.001), attending antenatal follow-ups ( β = 0.53; 95%CI 0.30–0.76; *P* = 0.001), and seeking information from healthcare providers (β = 0.93; 95%CI 0.60–1.25; *P* = 0.001). These findings underscore the urgent need for targeted educational campaigns, particularly in underserved and remote areas. Strengthening antenatal services and leveraging trusted information sources could enhance early recognition of danger signs and timely medical intervention, ultimately contributing to reduced neonatal mortality rates in Egypt.

**Supplementary Information:**

The online version contains supplementary material available at 10.1038/s41598-026-50756-z.

## Introduction

The neonatal period, encompassing the first 28 days of life, is critical for infant survival and development. It is also a key focus of Sustainable Development Goal (SDG) number 3 (Good Health and Well-Being), which aims to end preventable deaths of newborns and children under five years of age ^1^. Neonatal mortality continues to have a substantial global health challenge, constituting approximately 40% of under-five mortality and 57.0% of infant mortality worldwide. An estimated four million neonatal deaths occur annually, with the vast majority (99.0%) taking place in low- and middle-income countries. Nearly half of these deaths occur at home, highlighting persistent inequities in access to skilled birth attendance and essential neonatal care ^2^.

A major contributor to newborn morbidity and mortality is the delay in recognizing neonatal danger signs (NDSs). These signs include poor or no sucking, lethargy, difficulty breathing, abnormal body temperature, jaundice, umbilical cord bleeding, diarrhea, convulsions, and vomiting ^3–5^. The World Health Organization (WHO) asserts the importance of maternal knowledge of NDSs as a key strategy to decrease neonatal mortality ^6^. The WHO recommends focusing on nine primary danger signs: inability to feed, convulsions, rapid breathing (60 breaths or more per minute), chest indrawing, high fever (37.5 °C or more), hypothermia (35.4 °C or less), jaundice, lethargy or unconsciousness, and signs of local infection ^7^. However, researchers considered more symptoms as danger signs like dehydration ^8,9^ diarrhea, delayed meconium passage, and bloody stool ^10,11^. Therefore, mothers’ knowledge of these NDSs plays a vital role in facilitating early detection and prompt care-seeking behavior ^12,13^. A three-year interventional study in India demonstrated that enhanced maternal knowledge of danger signs correlated with a decrease in untreated neonatal symptoms ^14^.

Multiple factors influence a mother’s knowledge of NDSs, including age, education, occupational status, place of residence, antenatal care (ANC) and postnatal care (PNC) history, parity, and place of delivery ^3,15–18^. Bulto et al.,^19^ further identified maternal education, PNC follow-up, and place of delivery as significant predictors of mothers’ knowledge of NDSs. Understanding these factors is crucial for developing targeted interventions to improve maternal knowledge and, consequently, neonatal health outcomes. It is worth noting that the level of knowledge about these signs varies considerably across different countries and regions ^20 12,13,26with insifficient knowledge more pronounced in Low and Middle-income countries (LMIC) ^. For example, Abdulrida et al., ^21^ found that mothers’ knowledge of NDSs in Baghdad was insufficient, highlighting the need for targeted educational interventions.

In Egypt, the neonatal mortality rate was reduced from 33.22 deaths per 1,000 live birth in 1990 to 9.25 in 2024 ^5^. Although this reflects substantial progress, there is still room for improvement to meet national and global health targets ^22^. However, research on maternal knowledge of NDSs in the Egyptian context remains limited particularly across diverse geographical regions and varying healthcare system capacities. Degefa et al., ^18^ demonstrated that even in areas with high institutional delivery rates, mothers’ knowledge of NDSs could be suboptimal, emphasizing the need for continuous education.

 Therefore, this study aimed to evaluate maternal knowledge about NDSs across different regions of Egypt. Specifically, it addressed two key questions: first, what is the level of knowledge of NDSs among mothers in various regions of Egypt? Second, what are the determinants associated with mothers’ knowledge of these danger signs? The findings are expected to inform targeted interventions and policies to improve maternal education, promote early recognition of NDSs, and ultimately contribute to reducing neonatal mortality in Egypt.

## Methodology

### Study design, setting, and participants

A cross-sectional study was conducted from January to March 2025, in Egyptian public hospitals and primary healthcare facilities. The participants were recruited from mothers of infants aged between 1 and 24 months who attended these facilities to seek medical care or immunization during the data collection period. Mothers less than 18 years of age, those whose children had chronic diseases, or those who had mental or psychological illnesses precluding their participation were excluded.

### Sample size and sampling methods

The sample size was calculated using G* Power, based on the prevalence rate of good knowledge among the population in Alexandria, Egypt with 59.4% ^23^. Assuming a power of 95%, an α error = 0.05, effect size = 0.044, two-tailed analysis, the minimum required sample size was 1686 and rounded up to 1800 to compensate for a non-response rate of 5%. Regarding determinants of good knowledge, based on the prevalence of good knowledge among Ethiopian mothers who received information on NDSs 84.8% ^17^, Assuming a power of 95%, an α error = 0.05, effect size = 0.05, two-tailed analysis, the minimum required sample size was 580 and rounded up to 610 to compensate for a non-response rate of 5%. The effect size was calculated based on the results of the pilot study. We considered the larger sample size requirement to ensure sufficient statistical power. The design effect was not used. Although the study enrolled participants from several health facilities, the lack of probability-based cluster sampling and the expected minimal intra-cluster correlation were judged unlikely to meaningfully influence the required sample size.

**Fig. 1:**
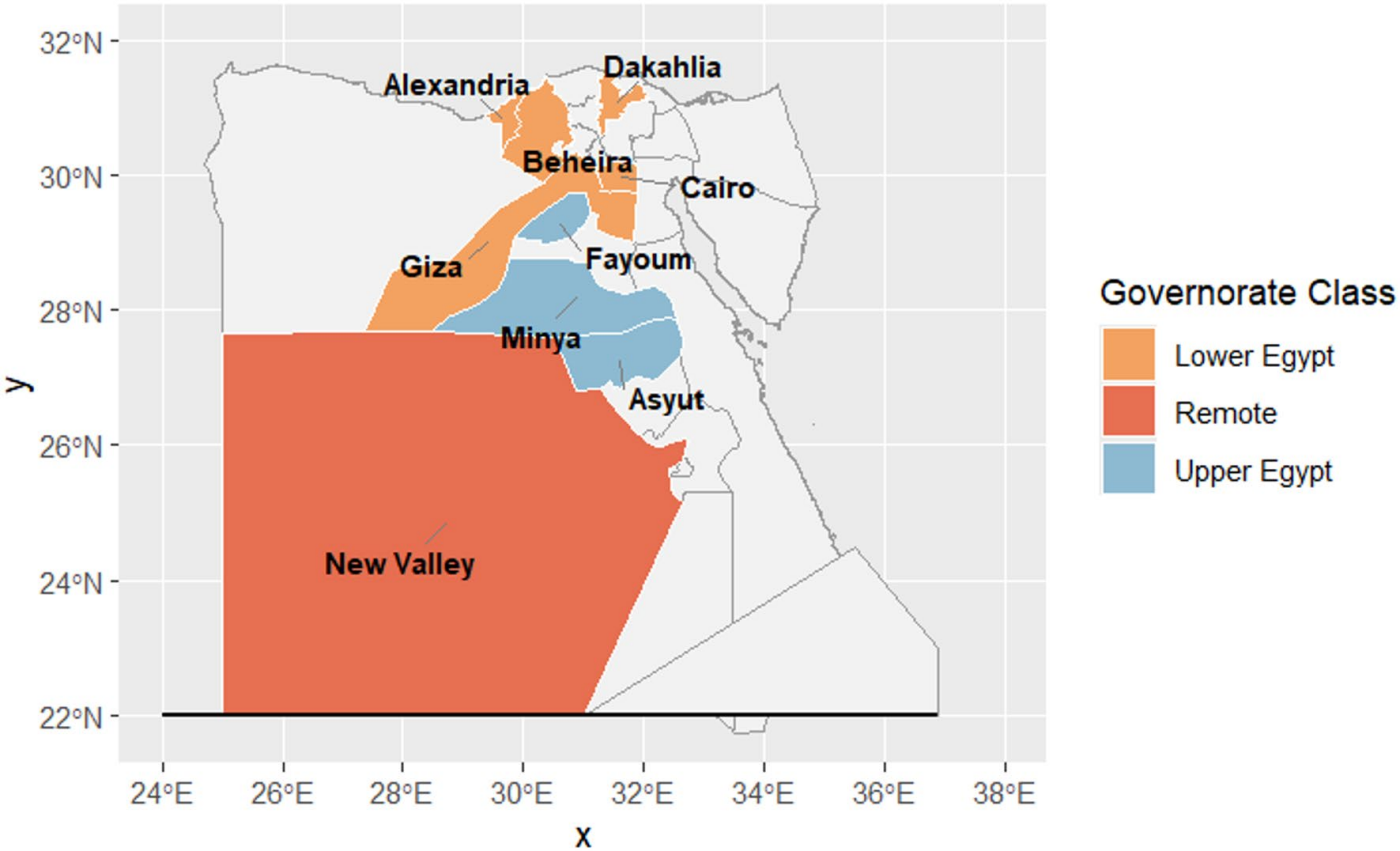
Geographic distribution of the study governorates across Egypt.

We used a multistage stratified sampling approach to improve representativeness across geographic regions, in which all governorates were listed and stratified into upper, lower, and remote Egypt. We randomly selected three governorates from Upper Egypt (Fayoum, Minya, and Asyut), five governorates from Lower Egypt (Cairo, Giza, Alexandria, Beheria, and Dakahlia), and one remote governorate (New Valley), Fig. 1. All selected governments were uncovered by the Universal Health Insurance (UHI) system. The sample was equally allocated across all selected governorates to ensure representativeness and minimize the impact of population density variations between highly populated and less populated areas. **Supplementary Table ****1** includes all sites of data collection and the population count of the selected governorates to according to the Central Agency for Public Mobilization and Statistics (CAPMAS) ^24^. Within each selected governorate, participants were recruited consecutively from predefined study settings until the required sample size was achieved. We followed the Checklist for Strengthening the Reporting of Observational studies in Epidemiology (STROBE) **Supplementary Table 2**^25^.

### Pilot study

Before data collection, we conducted a pilot study on 50 mothers to investigate the clarity of questions, the sequence of questions, the time to complete the survey, calculate the effect size, and response rate. Most of the questions were clear, except for a few minor edits. The prevalence of good knowledge was 55.0%. The response rate was 94% (47/50).

### Measurement and data collection

A structured interviewing questionnaire was used to collect data from participants through face-to-face interviews. The interview took 5–10 minutes by the research team in a separate room/space to ensure participants’ privacy. Before data collection, the interviewers received online educational sessions on data collection and the questionnaire. The questionnaire was divided into three sections, with the first section addressing socio-demographic characteristics of the participants including age, marital status (married, divorced, or widowed), family income (self-reported and categorized into (sufficient, sufficient and save, insufficient, or insufficient and take loans), educational level, and occupational status for both parents (categorized for mothers as employed or not, while categorized among their partners into causal work or unemployed, business owner, employee, or workers). The second section comprised the reproductive and maternity health care service characteristics (number of children, ANC utilization, place and mode of childbirth delivery, and paternal attendance of ANC and PNC visits. The third section included the validated Arabic Questionnaire to assess the Knowledge of Neonatal Danger Signs (AQ-KNDS) ^23^. It consists of 16 questions covering various NDSs that the participant should assess each sign as “dangerous”, “not dangerous,” or “do not know”. Scoring was as follows: one for “dangerous” and zero for “not dangerous” or “do not know” consistent with the original AQ-KNDS validation study, as both responses reflect lack of correct recognition of neonatal danger signs. All 16 items represent recognized dangerous signs, with maximum possible score of 16; therefore, higher scores indicate greater maternal knowledge.

### Statistical analysis

The statistical analysis was performed using R software (version 4.3). Continuous variables were assessed for normality using the Shapiro-Wilk test and visual inspection of histograms and Q-Q plots. Due to skewness, data were presented using median with interquartile range (IQR). Categorical variables were summarized as frequencies and percentages. For unadjusted comparisons of knowledge scores across categorical variables, Mann-Whitney U test was used for two-group comparisons, while Kruskal-Wallis test was employed for comparisons involving three or more groups, with statistical significance set at *p* < 0.05. Although knowledge in the AQ-KNDS was initially categorized into good and poor levels based on the median score, we constructed a multiple linear regression model to handle the limitations of dichotomizing continuous variables, including loss of information and the use of an arbitrary cut-off. Therefore, the knowledge score was analyzed as a continuous outcome using linear regression. Variables that demonstrated statistical significance in univariate analyses, along with clinically relevant variables identified a priori were included as candidate predictors. The final model included Governorate of residence, urbanity, family income, number of ANC follow-up visits, source of educational lectures, and preferred source of health information. Categorical variables were entered as factors with appropriate reference categories selected based on clinical relevance and the lowest observed knowledge scores in unadjusted analyses. Prior to interpreting the regression results, all key assumptions of linear regression were systematically evaluated. The linearity assumption was assessed using a residual versus fitted values plot with a Lowess smooth line, which demonstrated a random scatter of points around the zero line without systematic curvature, indicating that linearity was satisfied. The independence of errors was assessed using the Durbin-Watson test, which yielded a statistic of approximately 2.0 (within the acceptable range of 1.5–2.5), confirming no significant autocorrelation in the residuals, as expected given the cross-sectional study design. Homoscedasticity (constant variance of errors) was evaluated using the Breusch-Pagan test (*p* > 0.05) and visual inspection of the Scale-Location plot. The normality of residuals was assessed using the Q-Q plot. To address any potential concerns regarding standard errors, robust standard errors (heteroscedasticity-consistent estimators) were employed. Multicollinearity was assessed using the Variance Inflation Factor (VIF) with conventional threshold of 5–10, confirming no problematic multicollinearity among predictors. Influential observations were evaluated using Cook’s distance, with values below the threshold of 4/n, indicating that no single observation unduly influenced the regression coefficients and that the model results are stable and not driven by outliers. Global model validation was performed using the gvlma test, which confirmed overall acceptable model performance. Model fit was evaluated using the R-squared and adjusted R-squared values, representing the proportion of variance in the outcome explained by the model. The overall model significance was assessed using the F-test. Both crude (unadjusted) and adjusted regression results are reported to demonstrate the effect of confounding adjustment. Results are presented as unstandardized beta coefficients (β) with 95% confidence intervals (CI), and p-values. A sensitivity analysis was conducted excluding influential observations identified by Cook’s distance. The reduced model (excluding 94 observations, 5.2% of the sample) showed improved model fit (R² = 0.158 vs. 0.131; AIC = 6717 vs. 8253) and was therefore selected as the primary model for interpretation. All statistical tests were two-tailed, with a significance level of α = 0.05.

## Results

The study surveyed 1900 Egyptian mothers with 1831 participants included (response rate = 96.3%). Table 1 shows the sociodemographic characteristics of mothers and their partners. The age distribution reveals that almost half of the mothers (55.1%) fell within the 25–35 years age range, followed by 31.0% who were younger than 25 years. The median (IQR) of age was 27.0 [23.0–32.0]. Most mothers (97.3%) were married, 58.7% were living in urban areas, 73.8% had high school or lower degrees, and 84.2% were housewives. Financially, 58.4% reported that their family’s monthly income was sufficient. Regarding their intimate partners, 72.0% of fathers had high school education or lower degrees. In terms of occupation, 31.2% were workers, followed by employees (29.2%). The median (IQR) working hours for the fathers were 8.0 [8.0–12.0].

**Table 1:** Sociodemographic characteristics of the participating mothers and their partners (*N* = 1831).

Variables	Variable	Frequency *n* (%)
Age	Median (IQR)	27.0 [23.0–32.0]
Marital status	Married	1782 (97.3%)
	Divorced	35 (1.9%)
	Widow	14 (0.8%)
Residency	Alexandria	201 (11.0%)
	Asyut	201 (11.0%)
	Beheira	218 (11.9%)
	Cairo	203 (11.1%)
	Dakahlia	202 (11.0%)
	Fayoum	202 (11.0%)
	Giza	200 (10.9%)
	Minya	200(10.9%)
	New Valley	204 (11.1%)
Urbanity	Urban	1075 (58.7%)
	Rural	712 (38.9%)
	Remote area	44 (2.4%)
Maternal education	High school or below	1352 (73.8%)
	University and postgraduates	479 (26.2%)
Paternal educationl	High school or below	1318 (72.0%)
	University and postgraduates	513 (28.0%)
Mother’s employment status	Working	290 (15.8%)
	Housewife	1541 (84.2%)
Paternal working sector	Employee	542 (29.6%)
	Business owner	332 (18.1%)
	Worker	572 (31.2%)
	Casual Labor or Unemployed	385 (21.1%)
Family monthly income	Sufficient and save	141(7.7%)
	Sufficient	1069 (58.4%)
	Not sufficient	428 (23.4%)
	Not sufficient and loan	193(10.5%)
Working hours for father	Median (IQR)	8.0 [8.0–12.0]

Data are summarized using descriptive statistics. Categorical variables are reported as frequency and percentage [n (%)], while continuous variables are presented as median and interquartile range [Q1-Q3]. Rounding may result in minor discrepancies in percentage totals. Abbreviations: IQR, interquartile range; N, total number of participants (*N* = 1831).

Most mothers delivered in institutional settings, with 39.7% utilizing public hospitals or health units, while 36.5% delivered in private hospitals and 22.2% delivered in private clinics. Almost four-fifths of them (80.1%) had cesarean delivery. The median (IQR) for the number of children was 2.0 [2.0–3.0], while was 10.0 [5.0–17.0] for the age (in months) of the youngest infant. More than half of the infants (53.8%) were males. Regarding ANC, 70.5% of the mothers attended four or more follow-up visits, while 29.5% reported fewer visits or could not recall the number of visits they had attended. Paternal attendance at these visits was limited, with only 19.3% attending all scheduled visits, while 38.5% didn’t attend any follow-up visits or couldn’t remember the number of visits. Similarly, 27.4% of fathers attended all scheduled pediatric follow-up visits within the first month after the baby’s birth, while 49.8% did not attend any visits or were unsure of the number. About three-quarters (74.1%) of mothers reported that they didn’t receive any educational lectures about neonatal health, 16.4% received them at primary healthcare centers, and only 5.5% reported receiving them at public hospitals. Regarding the preferred information source on newborn health, 43.6% of mothers reported that they prefer to seek information regarding neonatal health from their healthcare providers, 29.8% preferred asking friends and family members, and 2.1% relied on Television and radio programs. Table 2.

**Table 2:** Maternal characteristics of the studied mothers.

Categories	Parameter	Frequency *n* (%)
Place of delivery	Home	29 (1.6%)
	A public hospital or primary healthcare center	726 (39.7%)
	Private clinic	407 (22.2%)
	Private hospital	669 (36.5%)
Type of delivery	Normal vaginal	365 (19.9%)
	Cesarean	1466 (80.1%)
Number of children	Median [Q1– Q3]	2.0 [2.0–3.0]
Age of the youngest infant in months	1–5	478 (26.1%)
	6–11	536 (26.3%)
	12–17	386 (21.1%)
	18–24	431(23.5%)
	Median [Q1– Q3]	10.0 [5.0–17.0]
Gender of the youngest infant	Male	985 (53.8%)
	Female	846 (46.2%)
Number of ANC follow-ups	Less than 4 visits or none	540 (29.5%)
	4 or more	1291 (70.5%)
Paternal attendance in ANC Visits	All scheduled visits	353 (19.3%)
	Limited number of visits (< 4)	772 (42.2%)
	Did not remember or none	706 (38.5%)
Paternalattendance in pediatric follow-up Visits in the first month:	Attended all scheduled visits	502 (27.4%)
	Attended a limited number of visits (≤ 2)	417 (22.8%)
	Did not remember or none	912 (49.8%)
Source of educational lectures on neonatal health	Public hospital	101 (5.5%)
	Primary healthcare center	300 (16.4%)
	Private clinic	73 (4.0%)
	None	1357 (74.1%)
Preferred information source on newborn health	TV & Radio	39 (2.1%)
	Family & friends	546 (29.8%)
	Healthcare providers	798 (43.6%)
	Internet	307 (16.8%)
	Social media	141 (7.7%)

Data are presented as frequency and percentage [n (%)]. Percentages may not sum to 100% due to rounding. Continuous variables are presented as median with interquartile range (IQR). Abbreviations: ANC, antenatal care; IQR, interquartile range; N, total sample size (*N* = 1831).

**Fig. 2:**
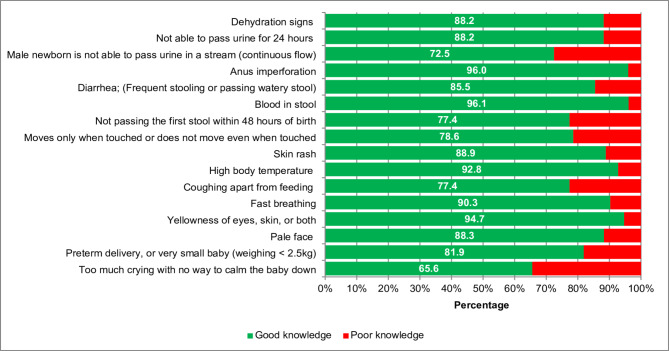
Prevalence of good knowledge of neonatal dangerous signs among the studied mothers.

**Fig. 3:**
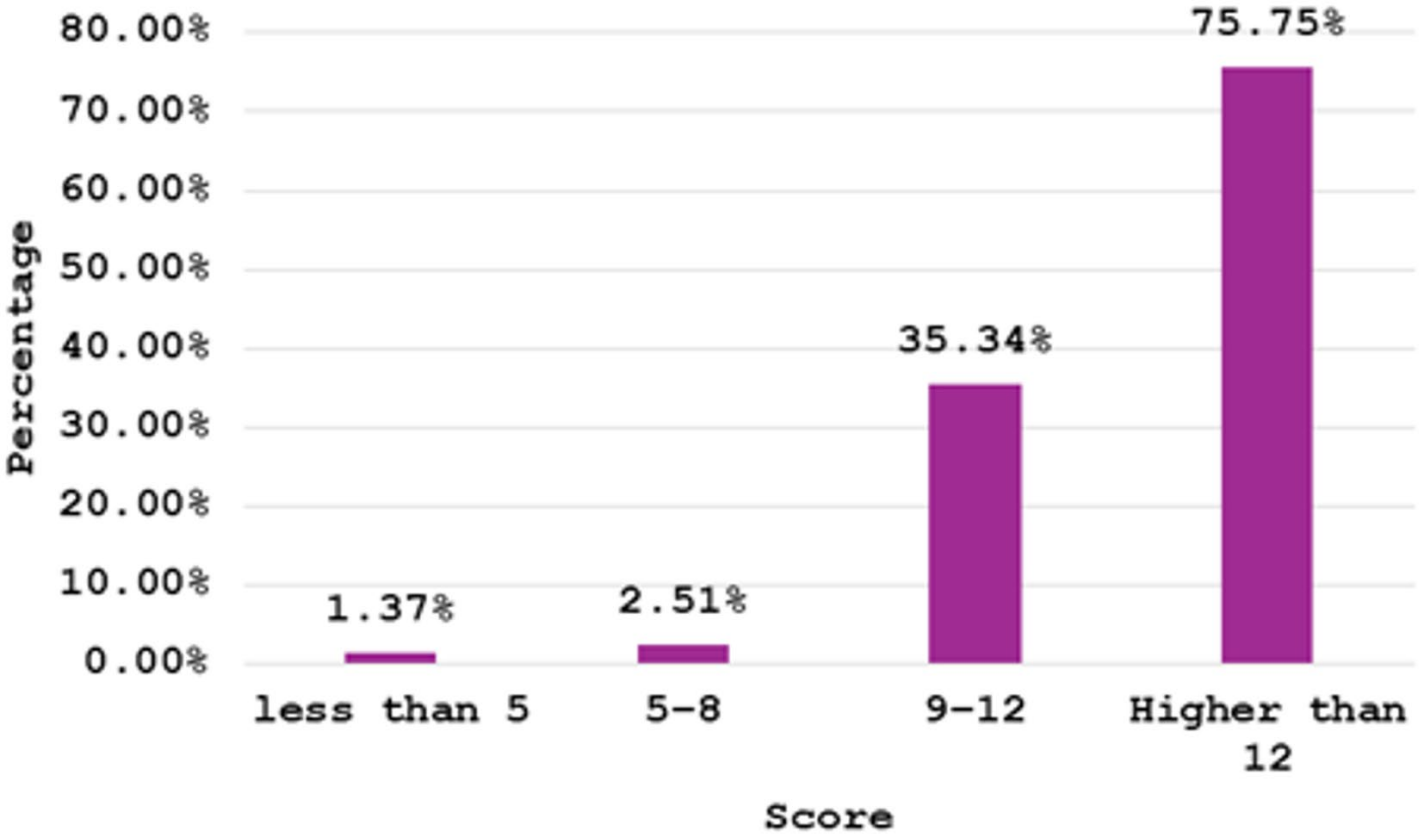
Distribution of knowledge scores among the studied mothers.

Cronbach’s Alpha for the questionnaire demonstrated acceptable reliability (α = 0.76, 95% CI [0.74, 0.77]; average inter-item correlation = 0.18), which indicates good internal consistency among the items on the scale. **Supplementary Tables 3**,**4**. Figure 2 illustrates the prevalence of good knowledge of NDSs among mothers. The most known dangerous sign was the presence of blood in stool (96.1%), while excessive crying was the least well-known dangerous sign, with 65.6%. **Supplementary Table 5** illustrates the distribution of good knowledge of NDSs among governments. Figure 3 illustrates the distribution of maternal knowledge scores. Approximately three-quarters of the Egyptian mothers achieved scores exceeding 75.0%, while about 20.0% obtained scores ranging between 50.0% and 75.0%.

Maternal knowledge scores regarding neonatal danger signs showed significant variation across several sociodemographic and health-related factors. Overall, the median knowledge score in the study population was 14.0 [IQR: 13.0–16.0]. Higher scores were observed among mothers with university education (median: 15.0 vs. 14.0, *p* = 0.006), employed mothers (15.0 vs. 14.0, *p* < 0.001), and those reporting sufficient or saving household income (median up to 16.0 vs. 14.0, *p* < 0.001). Similarly, mothers who attended ≥ 4 ANC visits had significantly higher scores compared with those who did not (15.0 vs. 14.0, *p* < 0.001). Exposure to educational lectures at private clinics was also associated with the highest median score (16.0, *p* < 0.001), and reliance on healthcare providers as the preferred information source yielded higher knowledge levels (median: 15.0 vs. 13.0–14.0 among other sources, *p* < 0.001). In contrast, no statistically significant differences were found for maternal age (median range: 14.0 across all groups, *p* = 0.237), marital status (*p* = 0.199), paternal education (*p* = 0.24), baby gender (*p* = 0.117), or most infant age categories (*p* = 0.628). Table 3.

**Table 3:** Factors associated with maternal knowledge score regarding neonatal danger signs among participating mothers (*N* = 1,831).

Variable	Category	*N* (%)	Score (Median, IQR)	Test Statistic	*P*-value
Governorate	Alexandria	201 (11.0)	14.0 (12.0–15.0)	120.5 †	< 0.001
	Asyut	201 (11.0)	14.0 (13.0–16.0)		
	Beheira	218 (11.9)	15.0 (13.3–16.0)		
	Cairo	203 (11.1)	14.0 (13.0–16.0)		
	Dakahlia	202 (11.0)	14.0 (13.0–16.0)		
	Fayoum	202 (11.0)	15.0 (13.0–16.0)		
	Giza	200 (10.9)	14.0 (12.0–15.0)		
	Minya	200 (10.9)	15.0 (12.0–16.0)		
	New Valley	204 (11.1)	13.0 (12.0–14.0)		
Urbanity	Rural	712 (38.9)	15.0 (13.0–16.0)	12.1 †	0.002
	Urban	1,075 (58.7)	14.0 (12.0–16.0)		
	Remote area	44 (2.4)	14.0 (11.0–16.0)		
Maternal age (years)	< 20	178 (9.7)	14.0 (12.0–16.0)	5.5 †	0.237
	20-<25	533 (29.1)	14.0 (12.0–16.0)		
	25-<30	586 (32.0)	14.0 (13.0–16.0)		
	30->35	359 (19.6)	14.0 (12.0–16.0)		
	≥ 35	175 (9.6)	14.0 (13.0–16.0)		
Marital tatus	Married	1,782 (97.3)	14.0 (13.0–16.0)	3.2 †	0.199
	Divorced	35 (1.9)	14.0 (12.0–15.0)		
	Widowed	14 (0.8)	15.0 (14.0–16.0)		
Maternal education	High school or lower	1,352 (73.8)	14.0 (13.0–16.0)	297,168 ‡	0.006
	University or higher	479 (26.2)	15.0 (13.0–16.0)		
Paternal education	High school or lower	1,318 (72.0)	14.0 (13.0–16.0)	326,369 ‡	0.24
	University or higher	513 (28.0)	14.0 (13.0–16.0)		
Maternal employment	Housewife	1,541 (84.2)	14.0 (12.0–16.0)	191,990 ‡	< 0.001
	Yes	290 (15.8)	15.0 (13.0–16.0)		
Paternal employment	Employee	542 (29.6)	14.0 (13.0–16.0)	35.2 †	< 0.001
	Worker	572 (31.2)	14.0 (13.0–16.0)		
	Business owner	332 (18.1)	14.0 (12.0–15.0)		
	Causal/ Unemployed	385 (21.1)	14.0 (12.0–15.0)		
Paternal work hours	≤‎ 10 h/day	1,206 (65.9)	14.0 (13.0–16.0)	1.9 †	0.379
	11–20 h/day	558 (30.5)	14.0 (13.0–16.0)		
	‎>‎20 h/day	67 (3.6)	14.0 (12.0–16.0)		
Number of children	1	422 (23.0)	14.0 (12.0–16.0)	5.1 †	0.28
	2	655 (35.8)	14.0 (13.0–16.0)		
	3	480 (26.2)	14.0 (12.8–16.0)		
	4	191 (10.4)	14.0 (13.0–16.0)		
	≥ 5	83 (4.6)	14.0 (12.5–16.0)		
Monthly family income	Insufficient	428 (23.4)	14.0 (12.0–15.0)	39.1 †	< 0.001
	Insufficient with loans	193 (10.5)	14.0 (12.0–16.0)		
	Sufficient	1,069 (58.4)	14.0 (13.0–16.0)		
	Sufficient and saving	141 (7.7)	16.0 (14.0–16.0)		
Baby gender	Male	985 (53.8)	14.0 (13.0–16.0)	399,305 ‡	0.117
	Female	846 (46.2)	14.0 (12.0–16.0)		
Baby age (months)	1–< 6 months	636 (34.7)	14.0 (12.0–16.0)	1.7 †	0.628
	6-–<12 months	565 (30.9)	14.0 (13.0–16.0)		
	12–<18 months	398 (21.7)	14.0 (13.0–16.0)		
	≥ 18 months	232 (12.7)	14.0 (13.0–16.0)		
	Governmental hospital/unit	726 (39.7)	14.0 (12.0–15.0)		
Place of delivery	Private hospital	669 (36.5)	14.0 (13.0–16.0)	39.1 †	< 0.001
	Private clinic	407 (22.2)	15.0 (13.0–16.0)		
	Home	29 (1.6)	15.0 (12.0–16.0)		
Delivery type	Cesarean section	1,466 (80.1)	14.0 (13.0–16.0)	284,868 ‡	0.051
	Normal vaginal delivery	365 (19.9)	14.0 (12.0–15.0)		
ANC follow-up visits	≥ 4 visits	1,291 (70.5)	15.0 (13.0–16.0)	410,734 ‡	< 0.001
	Less than 4 visits or none	540 (29.5)	14.0 (12.0–15.0)		
Father’s ANC attendance	All scheduled visits	353 (19.3)	14.0 (13.0–16.0)	3.3 †	0.194
	Limited visits (< 4)	772 (42.2)	14.0 (13.0–16.0)		
	Did not remember/none	706 (38.5)	14.0 (12.0–16.0)		
Father’s pediatric follow-up	Attended limited visits (≤ 2)	919 (50.2)	14.0 (13.0–16.0)	435,937 ‡	0.128
	Did not remember/none	912 (49.8)	14.0 (12.0–16.0)		
Source of educational lectures	None	1,357 (74.1)	14.0 (12.0–16.0)	48.7 †	< 0.001
	Primary healthcare center	300 (16.4)	14.5 (13.0–16.0)		
	Public hospital	101 (5.5)	14.0 (13.0–16.0)		
	Private clinic	73 (4.0)	16.0 (15.0–16.0)		
Preferred information source	Healthcare providers	798 (43.6)	15.0 (13.0–16.0)	75.9 †	< 0.001
	Family & friends	546 (29.8)	14.0 (12.0–15.0)		
	Internet	307 (16.8)	14.0 (13.0–16.0)		
	Social media	141 (7.7)	13.0 (12.0–14.0)		
	TV & Radio	39 (2.1)	14.0 (13.0–15.0)		

† Kruskal–Wallis test, ‡ Mann–Whitney U test (Wilcoxon rank-sum test), Data are presented as frequency (percentage) for categorical variables and median (interquartile range) for maternal knowledge scores. Statistical significance was set at *p* < 0.05.

In the adjusted multivariable model (excluding 94 influential observations), several factors demonstrated statistically significant associations with knowledge scores; Geographic variation was evident: compared to mothers from New Valley (reference group), residing in Beheira (β = 1.20; 95% CI: 0.75, 1.65), Fayoum (β = 1.17; 95% CI: 0.72, 1.63), Minya (β = 0.94; 95% CI: 0.46, 1.43), and Cairo (β = 0.84; 95% CI: 0.44, 1.42), was associated with higher knowledge scores, (*p* = 0.001). Regarding urbanity, rural residence was associated with significantly higher knowledge scores compared to urban residence (β = 0.44; 95% CI: 0.23, 0.66, *p* < 0.001). Family income played a significant role; participants with sufficient income, as well as those with sufficient income and savings, had higher scores compared to those with insufficient income (β = 0.32; 95% CI: 0.09, 0.54, *p* = 0.006) and (β = 0.49; 95% CI: 0.09, 0.89, *p* = 0.016) respectively. The categories of “insufficient with loans” did not show significant associations. Regarding healthcare utilization, having four or more ANC follow-up visits was significantly associated with higher knowledge scores compared to having no ANC visits (β = 0.53; 95% CI: 0.30, 0.76, *p* < 0.001), while place of delivery showed no significant impact. Regarding sources of education, receiving educational lectures from private clinics showed a strong positive association compared to receiving no lectures (β = 0.75; 95% CI: 0.26, 1.23, *p* = 0.003). Lectures from primary healthcare centers or public hospitals did not show significant associations. Concerning preferred information sources, compared to using social media, preferring healthcare providers as the primary information source was associated with significantly higher knowledge scores (β = 0.93; 95% CI: 0.60, 1.25, *p* < 0.001), as was using the internet (β = 0.64; 95% CI: 0.28, 1.00, *p* = 0.001) and family/friends (β = 0.48; 95% CI: 0.14, 0.82, *p* = 0.001). TV/radio as information sources did not show significant associations. Several variables showed no statistically significant associations in the reduced adjusted model, including: maternal age (all groups), parental education (all groups), paternal business ownership, family income categories (insufficient with loans, sufficient with savings), delivery at private hospitals or home, source of educational lectures from primary healthcare centers or public hospitals, and preferring family/friends or TV/radio as information sources. Table 4, **supplementary Tables 6**,** 7**,** supplementary Figs. 1–3.**

**Table 4:** Univariate and multivariable linear regression analysis of factors associated with neonatal danger signs knowledge score.

Variable	Level	Crude β (95% CI)	Crude *p*-value	Adjusted β (95% CI)	Adjusted *p*-value
Governorate (ref: New Valley)	Alexandria	0.60 (-0.12 to 1.09)	0.1	0.48 (0.05 to 0.91)	0.028*
	Asyut	0.69 (0.21 to 1.18)	0.005**	0.82 (0.40 to 1.24)	< 0.001***
	Beheira	1.19 (0.71 to 1.66)	< 0.001***	1.20 (0.75 to 1.65)	< 0.001***
	Cairo	0.60 (0.12 to 1.09)	0.014*	0.84 (0.44 to 1.24)	< 0.001***
	Dakahlia	0.44 (-0.04 to 0.92)	0.075	0.39 (-0.05 to 0.83)	0.081†
	Fayoum	0.74 (0.25 to 1.22)	0.003**	1.17 (0.72 to 1.63)	< 0.001***
	Giza	0.26 (-0.23 to 0.74)	0.299	0.62 (0.19 to 1.04)	0.005**
	Minya	-0.51 (-0.99 to -0.02)	0.040*	0.94 (0.46 to 1.43)	< 0.001***
Urbanity (ref: Urban)	Rural	0.99 (0.23 to 1.76)	0.011*	0.44 (0.23 to 0.66)	< 0.001***
	Remote area	0.60 (-0.16 to 1.36)	0.123	-0.11 (-0.78 to 0.57)	0.755
Mother age (ref: <20 years)	20–24 years	-0.16 (-0.59 to 0.27)	0.468	0.04 (-0.27 to 0.36)	0.789
	25–29 years	0.25 (-0.17 to 0.67)	0.246	0.26 (-0.06 to 0.57)	0.112
	30–34 years	0.03 (-0.43 to 0.48)	0.912	0.08 (-0.26 to 0.43)	0.636
	≥ 35 years	0.47 (-0.05 to 1.00)	0.078	0.39 (-0.01 to 0.79)	0.056†
Marital atatus (ref: Divorced)	Married	0.47 (-0.38 to 1.31)	0.28	—	—
	Widow	1.20 (-0.37 to 2.77)	0.133	0.77 (-0.32 to 1.86)	0.167
Maternal education (ref: High school or lower)	University or higher	0.24 (-0.03 to 0.50)	0.08	-0.11 (-0.36 to 0.15)	0.415
Paternal education (ref: High school or lower)	University or higher	0.11 (-0.15 to 0.37)	0.403	—	—
Maternal employment (ref: No)	Employed	0.56 (0.24 to 0.87)	< 0.001***	0.22 (-0.04 to 0.47)	0.1
Paternal employment sector (ref: Causal/unemployed)	Business owner	-0.28 (-0.64 to 0.09)	0.138	0.05 (-0.24 to 0.34)	0.741
	Employee	0.66 (0.32 to 1.00)	< 0.001***	0.17 (-0.11 to 0.46)	0.231
	Worker	0.52 (0.19 to 0.86)	0.002**	0.21 (-0.03 to 0.45)	0.090†
Paternal work hours (ref: ‎ ≤‎10 h)	11-20 h/day	-0.13 (-0.39 to 0.12)	0.303	—	—
	>‎20 h/day	-0.16 (-0.78 to 0.47)	0.625	—	—
Number of children (ref: 1 child)	2	0.20 (-0.11 to 0.51)	0.203	—	—
	3	0.01 (-0.32 to 0.34)	0.953	—	—
	4	0.25 (-0.18 to 0.69)	0.251	—	—
	≥ 5	0.00 (-0.59 to 0.60)	0.999	—	—
Monthly family income (ref: Insufficient)	Insufficient & take loans	-0.17 (-0.59 to 0.26)	0.443	-0.06 (-0.38 to 0.27)	0.742
	Sufficient	0.48 (0.20 to 0.76)	< 0.001***	0.32 (0.09 to 0.54)	0.006**
	Sufficient and save	0.75 (0.27 to 1.23)	0.002**	0.49 (0.09 to 0.89)	0.016*
Baby gender (ref: Female)	Male	0.19 (-0.04 to 0.43)	0.1	0.14 (-0.03 to 0.30)	0.107
Baby age (ref: 1-6 months)	6–˂12months	0.13 (-0.16 to 0.41)	0.383	0.09 (-0.12 to 0.30)	0.405
	12–˂18 months	0.19 (-0.13 to 0.51)	0.239	0.09 (-0.15 to 0.32)	0.477
	≥18–24 months	0.39 (0.01 to 0.77)	0.046*	0.14 (-0.14 to 0.42)	0.32
Place of delivery (ref: Government hospital)	Private hospital	0.54 (0.28 to 0.81)	< 0.001***	0.05 (-0.18 to 0.27)	0.683
	Private clinic	0.42 (0.12 to 0.73)	0.007**	0.22 (-0.06 to 0.51)	0.123
	Home	-0.02 (-0.95 to 0.91)	0.966	0.67 (-0.08 to 1.43)	0.081†
Delivery type (ref: Normal vaginal delivery)	Cesarean section	-0.15 (-0.44 to 0.14)	0.306	—	—
ANC visits (ref: Less than 4 visits or none)	≥ 4 visits	0.54 (0.29 to 0.80)	< 0.001***	0.53 (0.30 to 0.76)	< 0.001***
PaternalANC attendance (ref: All visits)	Limited visits (< 4)	0.25 (-0.07 to 0.57)	0.119	0.08 (-0.16 to 0.31)	0.514
	Did not remember/none	-0.13 (-0.45 to 0.19)	0.428	-0.05 (-0.32 to 0.23)	0.739
Paternal pediatric attendance (ref: All visits)	Limited visits (≤ 2)	—	—	—	—
	Did not remember/none	-0.40 (-0.63 to -0.17)	< 0.001***	-0.07 (-0.27 to 0.12)	0.470
Education source (ref: None)	Primary healthcare center	0.50 (0.18 to 0.81)	0.002**	-0.08 (-0.36 to 0.20)	0.576
	Private clinic	1.42 (0.83 to 2.01)	< 0.001***	0.75 (0.26 to 1.23)	0.003**
	Public hospital	0.47 (-0.03 to 0.98)	0.067	-0.14 (-0.55 to 0.28)	0.518
Preferred information source (ref: Social media)	Family & friends	0.34 (-0.01 to 0.69)	0.059	0.48 (0.14 to 0.82)	0.006**
	Internet	0.50 (0.01 to 0.99)	0.046*	0.64 (0.28 to 1.00)	< 0.001***
	Healthcare providers	0.80 (0.53 to 1.07)	< 0.001***	0.93 (0.60 to 1.25)	< 0.001***
	TV & Radio	-0.33 (-1.14 to 0.48)	0.423	0.56 (-0.12 to 1.24)	0.106

The multivariable linear regression model was statistically significant (F = 7.59, *p* < 0.001) and explained 15.8% of the variance in maternal knowledge scores (R² = 0.158, adjusted R² = 0.137). The residual standard error was 1.72, and the model AIC and BIC were 6717.13 and 6951.04, respectively. Significance codes for individual predictors: *** *p* < 0.001; ** *p* < 0.01; * *p* < 0.05; † *p* < 0.10. Variables with “—” in the adjusted model were excluded from multivariable analysis due to *p* ≥ 0.20 in univariate screening (Paternal education, Paternal work hours, Number of children, Delivery type) or because the variable was not significant in univariate analysis (Marital Status was included only in the reduced model based on the widow level *p* = 0.133).

## Discussion

The primary objective of this study was to assess the level of maternal knowledge regarding NDSs across various regions of Egypt and to identify determinants including socio-demographic factors associated with this knowledge. The study revealed that three-quarters of Egyptian mothers scored above 75.0% of AQ-KNDS score indicating good knowledge. The median knowledge score was 14.0 [IQR: 13.0–16.0]. Key findings revealed significant variations in knowledge based on geographical location. Several factors were significantly associated with better maternal knowledge, including, family income, attending four or more ANC visits, and receiving educational sessions at private clinics. Additionally, mothers who preferred to seek information from healthcare providers, family and friends, or the internet showed significantly better knowledge about NDSs.

### Interpretation of the main study findings

The participants studied showed an accepted level of knowledge about NDSs. This level of knowledge was comparable to the findings of a study conducted in Ethiopia revealed that post-discharge from neonatal intensive care unit (NICU), women had a prevalence of correct knowledge of 61.7% ^17^ and that conducted among 377 Palestinian mothers attending health centers for child vaccination, which revealed a level of knowledge of 51.0% ^12^. However, it is slightly higher than a good knowledge prevalence of  39.0% reported in an earlier study ^26^. Furthermore, our findings surpass those reported by Wudu et al., ^27^ who found that only 36.6% of participants were knowledgeable about NDSs. Moreover, the prevalence of good knowledge among 112  Chinese mothers attending an immunization setting in a rural setting was 42.0% ^20^. Low level of knowledge was reported in other countries as well, Madagascar ^28^, Ethiopia ^29,30^, Saudi Arabia ^31^.   This indicates some variability in knowledge levels across different populations and periods. These variations could indicate disparities in health education initiatives, availability of maternal and child health services, and the success of community outreach efforts. Furthermore, differences in study designs, including the use of different assessment tools, the targeting of specific populations (e.g., fathers only), or conducting assessments during certain time frames (e.g., perinatal or postpartum) may also play a role in the observed differences in knowledge levels. However, the fact that more than half of the participants in all the above-mentioned studies lacked adequate knowledge ^32,33^ underscores the need for enhanced public health interventions. Improving maternal awareness of NDSs is crucial for early recognition of life-threatening conditions and timely care-seeking behavior. Targeted educational campaigns, particularly during ANC and PNC visits, could play a vital role in bridging this knowledge gap and ultimately reducing neonatal morbidity and mortality.

Specifically, the lowest scores for good knowledge were observed in response to the following NDSs: “excessive crying with no way to calm the baby” and “male newborn unable to pass urine in a stream.” In contrast, the highest levels of awareness were reported for signs such as “anus imperforation” and “yellowish eyes, skin, or both.” These findings suggest that caregivers are more likely to recognize visible or well-known danger signs, such as jaundice or congenital anomalies, while subtle or less discussed signs, like persistent inconsolable crying or urinary abnormalities, are often overlooked. This highlights the need to broaden the scope of health education initiatives to include less recognized yet clinically significant symptoms. Emphasizing these signs during routine maternal and newborn health counseling could enhance early detection and prompt intervention, ultimately improving neonatal health outcomes. Other studies identified jaundice, hypothermia, and unable to breastfeed are the highest identified NDSs ^27,28,31^. On the other hand, yellowish discoloration, convulsion, and lethargy ^34,35^, poor feeding, excessive crying, eye-draining pus ^20,31^ were the least identified in other studies. This underscores the importance of comprehensive maternal education that emphasize not only the well-known danger signs but also those that are subtle yet clinically significant. Integrating such topics into routine ANC and PNC counseling, community outreach, and media-based awareness campaigns could greatly enhance early recognition and timely health-seeking behavior, ultimately improving neonatal survival and well-being.

Despite the recent national initiatives to enhance health system capacity, almost 75.0% of mothers mentioned they didn’t attend educational lectures regarding neonatal health and about only 40.0% of them seek health information from healthcare providers. The Egyptian health system is undergoing significant reform, with policies aimed at expanding coverage, quality, and access through primary care strengthening and the introduction of the UHI system emphasizing primary health care units as the backbone for preventive services and equitable care delivery. Conduction of the study in regions where the UHI system has not yet been implemented could interpret the observed gaps in maternal exposure to neonatal health education and limited engagement with formal health information sources. Further research could be conducted to assess the impact of UHI implementation on maternal knowledge by comparing regions with and without system adoption.

### Factors associated with good knowledge about NDSs

Sociodemographic factors: Maternal age was significantly associated with maternal knowledge of newborn danger signs in univariate not in multivariable analyses. This finding is consistent with most previous studies, which also reported no significant relationship between maternal age and knowledge levels. However, contrasting evidence was reported by Zhou et al., ^20^ who found that mothers under the age of 25 had 3.8 times higher odds of having poor knowledge compared to older mothers. This discrepancy may reflect contextual differences in education, access to healthcare information, or cultural factors influencing health literacy among younger mothers.

Governorates such as Fayoum and Beheira reported the highest levels of maternal knowledge regarding newborn danger signs, whereas the New Valley governorate had the lowest. Moreover, residence in the rural area significantly increased the level of knowledge. This geographic disparity may reflect differences in healthcare infrastructure, the availability and quality of maternal education programs, and access to ANC and PNC services ^36–38^.

In multivariable analysis, the number of ANC visits was a significant predictor for maternal knowledge. Similar findings were reported in multiple studies ^15,17,20,39^. That’s highlights importance of promoting ANC utilization, encouraging mothers to attend regular visits, and expanding access to healthcare services, particularly in underserved and remote areas.

Although receiving educational sessions at private clinics was associated with higher maternal knowledge, this finding should be interpreted with caution. It is likely influenced by underlying socioeconomic factors, as mothers attending private healthcare facilities may have higher education levels, better financial status, and greater access to health information. Similarly, poor maternal knowledge regarding infant feeding among Rwandan mothers was associated with lower socioeconomic status ^40^.

These findings highlight the critical role of in improving maternal awareness. Therefore, targeted interventions—particularly in low-performing areas like New Valley are essential to bridge the knowledge gap and promote equitable neonatal health outcomes.

### Implications of the study

Strengthening ANC promoting paternal involvement, and leveraging trusted information sources such as healthcare providers and digital platforms could further enhance maternal knowledge. Addressing socioeconomic factors like income and education is also crucial, as sufficient family income was a predictor of better maternal knowledge. By improving maternal education and awareness particularly in remote areas, early recognition of NDSs can be enhanced, leading to timely medical intervention and ultimately reducing neonatal mortality rates in Egypt.

### Strengths and limitations

The strengths of the study include its comprehensive geographic representation, large sample size, and use of a validated measurement tool (AQ-KNDS). The stratified sampling technique ensured representation across Upper Egypt, Lower Egypt, and remote areas, minimizing biases related to population density. However, limitations were noted, including the cross-sectional design, which restricted causal inferences, and reliance on self-reported data, which may have introduced recall or social desirability bias. Moreover, the study built on a quantitative analysis and did not explore the underlying reasons for poor maternal knowledge, such as cultural beliefs, social barriers, or misconceptions. Future research using qualitative methods could better understand these factors.

## Conclusions

The study on maternal knowledge of NDSs in Egypt provides critical insights into the factors influencing awareness and highlights areas for targeted interventions to improve neonatal health outcomes. The findings revealed that Egyptian mothers demonstrated good knowledge of NDSs, with significant disparities based on geographic location, income, and healthcare exposure. Mothers from Behera and Fayoum exhibited the highest levels of awareness, while those from New Valley showed the poorest knowledge score. Key predictors of better maternal knowledge included sufficient family income, attending four or more ANC visits, receiving educational sessions at private clinics, and relying on healthcare providers, family and friends, or the internet for information. These findings underscore the need for tailored educational interventions, particularly in underserved and remote areas, to bridge gaps in maternal awareness of NDSs. Strengthening ANC and PNC services, promoting paternal involvement, and leveraging trusted information sources such as healthcare providers and digital platforms could further enhance maternal knowledge. By addressing these gaps, policymakers and healthcare providers can empower mothers to recognize danger signs early, seek timely medical care, and ultimately contribute to reducing neonatal mortality rates in Egypt. This study adds valuable context-specific evidence to the global effort to improve neonatal health and survival.

## Supplementary Information

Below is the link to the electronic supplementary material.

Supplementary Material 1

## Data Availability

The datasets generated during this study are not publicly available due to ethical ‎restrictions and the inclusion of sensitive participant information. Data may be ‎available from the corresponding author upon reasonable request and with ‎approval from the institutional ethics committee.‎.
