# Supplementary material for: Assessing maternal knowledge of neonatal danger signs in Egypt: a cross-sectional study
Source: Sci Rep. 2026 May 8;16:14677. doi: 10.1038/s41598-026-50756-z (PMC13156303; doi:10.1038/s41598-026-50756-z)
Supplement: Supplementary file 1 — Supplementary Material 1 [file 41598_2026_50756_MOESM1_ESM.docx]

| **Supplementary table 1: Population count and distribution of centers of data collection in the selected Egyptian Governorates** | | |
| --- | --- | --- |
| **Government** | **Population count** | **Healthcare facilities** |
| **Alexandria** | 5,665,469 | - **El-Anfoushy Pediatric Hospital** - **El-Raml Pediatric Hospital** |
| **Beheira** | 7,141,185 | - **Kafr El-Dawar General Hospital** |
| **Giza** | 9,827,999 | - **El-Badrasheen Hospital** - **Tahrir General Hospital** - El-Badrasheen Health Center - Kafr Nassar Health Center - Kaabish Health Office |
| **Dakahlia** | 7,242,244 | - Sherbin Health Office - **Sherbin Central Hospital** - **Dikirnis General Hospital** |
| **Fayoum** | 4,271,403 | - Al-Hadaka Medical Center - Kaiman Fares Medical Center - High Dam Medical Center - **Fayoum General Hospital** |
| **Cairo** | 10,489,280 | - **Al-Galaa Teaching Hospital** |
| **El Minya** | 6,644,997 | - Qafada Health Office – Maghagha - Samalout Health Office - El-Adwa Health Office - Bani Mazar Health Office |
| **New Valley** | 276,423 | - **El-Kharga Hospital** |
| **Asyut** | 5,326,403 | - Walidiya Health Center - **Asyut General Hospital** - Maternity and Children's Hospital in Asyut - Ferial Childcare Center |

| **Supplemental Table 2: STROBE Statement - checklist for our study** | | | | |
| --- | --- | --- | --- | --- |
| Strobe recommendation | **Item No** | | **Our study** | **Page No** |
| **Title and abstract** | | | | |
| (*a*) Indicate the study’s design with a commonly used term in the title or the abstract | 1 | | (a)  Title: Assessing maternal knowledge of neonatal danger signs in Egypt: a cross-sectional study  Abstract: Study design included in abstract:  A cross-sectional study was conducted in healthcare ‎facilities‎ through face-to-face interviews from January to March 2025‎. | Page 2 |
| (*b*) Provide in the abstract an informative and balanced summary of what was done and what was found |  |  | (b) Structured abstract provided. | Page 2 |
| **Introduction** | | | | |
| Background/rationale:  Explain the scientific background and rationale for the investigation being reported | 2 | | Background outlined in the introduction section. Neonatal mortality remains a major global challenge, with delayed recognition of neonatal danger signs contributing significantly to increased morbidity and mortality. Maternal knowledge plays a vital role in early detection and timely care‑seeking, and it is influenced by factors such as maternal age, educational level, and access to antenatal and postnatal care services. Assessing maternal knowledge and identifying the factors that influence it are crucial for developing targeted interventions that strengthen maternal awareness and ultimately improve neonatal health outcomes. | Page 2-3 |
| Objectives:  State specific objectives, including any prespecified hypotheses | 3 | | Objective and hypothesis clearly stated in text:  This study aimed to evaluate maternal knowledge about NDSs across different regions of Egypt. Specifically, it addressed two key questions: first, what is the level of knowledge of NDSs among mothers in various regions of Egypt? Second, what factors are associated with mothers’ knowledge of these danger signs? The findings are expected to inform targeted interventions and policies to improve maternal education, promote early recognition of NDSs, and ultimately contribute to reducing neonatal mortality in Egypt. | Page 3 |
| **Methods** | | | |  |
| Study design:  Present key elements of study design early in the paper | 4 | | Described  “Key elements of the study included in the method: Study design, setting, participants, inclusion and exclusion criteria, sample size, sampling method, measurement tools, data collection, and statistical analysis. | Page 4-6 |
| Setting: Describe the setting, locations, and relevant dates, including periods of recruitment, exposure, follow-up, and data collection | 5 | | Described in Study design, setting, and participants and in measurement and data collection section | page 4&5 |
| Participants:  (a) Give the eligibility criteria, and the sources and methods of selection of participants.  (b) For matched studies, give matching criteria and the number of controls per case | 6 | | (a) Sample described.  The participants were recruited from mothers of infants aged up to 24 months who attended these facilities to seek medical care or immunization in the selected setting during the data collection period. Mothers less than 18 years of age, those whose children had chronic diseases, or those who had mental or psychological illnesses precluding their participation were excluded.  Method of selection described in Sample size and sampling methods section  (b) N/A | Page 4 |
| Variables:  Clearly define all outcomes, exposures, predictors, potential confounders, and effect modifiers. Give diagnostic criteria, if applicable | 7 | | Definitions of all variables, outcomes, and exposures are clearly defined in the Measurement and data collection section | Page 5 |
| Data sources/ measurement:  For each variable of interest, give sources of data and details of methods of assessment (measurement). Describe comparability of assessment methods if there is more than one group | 8* | | Source:  Described in text of Measurement and data collection section  A structured interviewing questionnaire consist of 3 section, , with the first section addressing socio-demographic characteristics of the participants, The second section comprised the reproductive and maternity health care service characteristics, The third section included the validated Arabic Questionnaire to assess the Knowledge of Neonatal Danger Signs (AQ-KNDS) | Page 5 |
| Bias:  Describe any efforts to address potential sources of bias | 9 | | Described in methods.  multistage stratified sampling approach was used to improve representativeness across geographic regions,  “The study employed a pilot-tested questionnaire, Before data collection, a pilot study was conducted on 50 mothers to investigate the clarity of questions, the sequence of questions, the time to complete the survey, calculate the effect size, and response rate. Before data collection, the interviewers received online educational sessions on data collection and the questionnaire. | Page 5-8 |
| Study size:  Explain how the study size was arrived at | 10 | | Described. | Page 4 |
| Quantitative variables:  Explain how quantitative variables were handled in the analyses. If applicable, describe which groupings were chosen and why | 11 | | Described, Statistical methods section.  Continuous variables were assessed for normality using the Shapiro-Wilk test and visual inspection of histograms and Q-Q plots. Due to skewness, data were presented using median with interquartile range (IQR). | Page 5 |
| Statistical methods:  a) Describe all statistical  methods, including those  used to control for  confounding  (*b*) Describe any methods used to examine subgroups and interactions  (*c*) Explain how missing data were addressed  (*d*) *Cross-sectional study*—If applicable, describe analytical methods taking account of sampling strategy  (*e*) Describe any sensitivity analyses | 12 | | (a) Described:  Due to skewness, quantitative data were presented using median with interquartile range (IQR). Categorical variables were summarized as frequencies and percentages. For unadjusted comparisons of knowledge scores across categorical variables. Mann-Whitney U test was used for two-group comparisons, while Kruskal-Wallis test was employed for comparisons involving three or more groups, with statistical significance set at p < 0.05. The knowledge score was analyzed as a continuous outcome using linear regression. All key assumptions of linear regression were systematically evaluated.  (b) N/A  (c) N/A  (d) We used a multistage stratified sampling approach to improve representativeness across geographic regions, in which all governorates were listed and stratified into upper, lower, and remote Egypt. We randomly selected three governorates from Upper Egypt (Fayoum, Minya, and Asyut), five governorates from Lower Egypt (Cairo, Giza, Alexandria, Beheria, and Dakahlia), and one remote governorate (New Valley),  (e) A sensitivity analysis was conducted excluding influential observations identified by Cook's distance (threshold = 4/n). | Page 5&6 |
| **Results** | | | | |
| Participants  (a) Report numbers of individuals at each stage of study—eg numbers potentially eligible, examined for eligibility, confirmed eligible, included in the study, completing follow-up, and analysed  (b) The outcome and the main exposure were available for  all participants.  (c) Consider use of a flow diagram | | 13 | (a) Described The study surveyed 1900 Egyptian mothers with 1831 participants included (rate 96.3%).  (b) available in table 1 :Sociodemographic characteristics of the participating mothers and their partners  Table 2: Maternal characteristics of the studied mothers‎  figure 2: Prevalence of good knowledge of neonatal dangerous signs among the studied mothers  (c) N/A. | Result section Page 7-10 |
| Descriptive data:  (a) Give characteristics of study participants (e.g. demographics, clinical, social) and information on exposures and potential  confounders  (b) Indicate the number of  participants with missing  data for each variable of  interest | | 14 | (a) Described.  (b) N/A | Page 7-9 |
| Outcome data:  Report numbers in each  exposure category, or  summary measures of  exposure | | 15 | These outcome data are reported in the Results section Table 3: Factors associated with maternal knowledge score regarding neonatal danger signs among participating mothers | Page 11-14 |
| Main results:  (a) Give unadjusted estimates and, if applicable, confounder adjusted estimates and their precision (eg, 95% confidence interval). Make clear which confounders were adjusted for and why they were included.  (b) Report category boundaries when continuous variables were categorized  (c) If relevant, consider  translating estimates of  relative risk into absolute  risk for a meaningful time  period | | 16 | (a) Added in table 4: Univariate and Multivariable Linear Regression Analysis of Factors Associated with Neonatal Danger Signs Knowledge Score  (b) Added in table 1-4  (c) N/A | Page 15-17 |
| Other analysis:  Report other analyses  done-e.g. analyses of  subgroups and interactions, and sensitivity  analyses | | 17 | Sensitivity analysis using Cook's distance measured the influence of each observation on the regression coefficients by quantifying the change in predicted values when that observation is excluded from the model. | Page 28 |
| **Discussion** | | | | |
| Key results:  Summarize key results with reference to study objectives. | 18 | | Done | Page 17 |
| Limitations:  Discuss limitations of the study, taking into account sources of potential bias or imprecision.  Discuss both direction and magnitude of  any potential bias | 19 | | Limitations were noted, including the cross-sectional design, which restricted causal inferences, and reliance on self-reported data, which may have introduced recall or social desirability bias. | Page 20 |
| Interpretation:  Give a cautious overall interpretation of results considering objectives, limitations, multiplicity of analyses, results from similar studies, and other relevant evidence. | 20 | | Mentioned in Interpretation of the main study findings & Strengths and limitations sections. | Page 17-20 |
| Generalisability:  Discuss the generalizability  (external validity) of the  study results | 21 | | The strengths of the study include its comprehensive geographic representation, large sample size, and use of a validated measurement tool (AQ-KNDS). The stratified sampling technique ensured representation across Upper Egypt, Lower Egypt, and remote areas, minimizing biases related to population density. | Page 20 |
| Funding:  Give the source of funding and the role of the funders for the present study and, if applicable, for the original study on which the present article is based | 22 | | Funding: This research received no external funding.  The authors declare no competing interests. | Page 22 |

| **Supplementary table 3. Internal Consistency Reliability Statistics** | |
| --- | --- |
| **Reliability Index** | **Estimate** |
| **Cronbach's α (raw)** | 0.76 |
| **Cronbach's α (standardized)** | 0.78 |
| **Guttman's Lambda 6** | 0.78 |
| **Average inter-item correlation** | 0.18 |
| **Signal-to-Noise ratio** | 3.5 |
| **95% CI (Feldt)** | 0.74–0.77 |
| **Asymptotic SE** | 0.008 |
| **Mean item score** | 0.86 |
| **SD of item scores** | 0.16 |

| **Supplementary table 4: Reliability if Individual Items Are Deleted** | | | |
| --- | --- | --- | --- |
| **Item** | **α if deleted** | **Std. α if deleted** | **Item-Total r** |
| Q1 | 0.74 | 0.77 | 0.38 |
| Q2 | 0.75 | 0.77 | 0.32 |
| Q3 | 0.75 | 0.77 | 0.34 |
| Q4 | 0.75 | 0.77 | 0.35 |
| Q5 | 0.74 | 0.77 | 0.37 |
| Q6 | 0.74 | 0.77 | 0.36 |
| Q7 | 0.75 | 0.77 | 0.32 |
| Q8 | 0.74 | 0.76 | 0.39 |
| Q9 | 0.74 | 0.77 | 0.36 |
| Q10 | 0.75 | 0.77 | 0.35 |
| Q11 | 0.74 | 0.76 | 0.41 |
| Q12 | 0.74 | 0.77 | 0.34 |
| Q13 | 0.74 | 0.76 | 0.41 |
| Q14 | 0.75 | 0.77 | 0.34 |
| Q15 | 0.74 | 0.76 | 0.41 |
| Q16 | 0.74 | 0.76 | 0.39 |

| **Supplementary table 5: Distribution of Knowledge of neonatal danger signs by governments** | | | | | | | | | |
| --- | --- | --- | --- | --- | --- | --- | --- | --- | --- |
| **Characteristic** | **Alexandria**  N = 201^1^ | **Asyut**  N = 201^1^ | **Beheira**  N = 218^1^ | **Cairo**  N = 203^1^ | **Dakahlia**  N = 202^1^ | **Fayoum**  N = 202^1^ | **Giza**  N = 200^1^ | **Minya**  N = 200^1^ | **New Valley**  N = 204^1^ |
| Excessive crying with no way to calm the baby down | 132 (66%) | 169 (84%) | 161 (74%) | 139 (68%) | 141 (70%) | 149 (74%) | 98 (49%) | 140 (70%) | 73 (36%) |
| Premature delivery (before 37weeks gestational age), or birth weight < 2.5kg | 143 (71%) | 168 (84%) | 140 (64%) | 175 (86%) | 170 (84%) | 174 (86%) | 169 (85%) | 158 (79%) | 202 (99%) |
| Pale face | 183 (91%) | 173 (86%) | 190 (87%) | 191 (94%) | 180 (89%) | 180 (89%) | 167 (84%) | 156 (78%) | 196 (96%) |
| Yellowish discoloration of skin, sclera or both | 196 (98%) | 197 (98%) | 215 (99%) | 193 (95%) | 196 (97%) | 188 (93%) | 188 (94%) | 173 (87%) | 188 (92%) |
| Fast breathing | 191 (95%) | 170 (85%) | 208 (95%) | 192 (95%) | 178 (88%) | 177 (88%) | 189 (95%) | 166 (83%) | 182 (89%) |
| Coughing apart from feeding | 148 (74%) | 174 (87%) | 187 (86%) | 146 (72%) | 150 (74%) | 139 (69%) | 142 (71%) | 154 (77%) | 178 (87%) |
| High body temperature | 184 (92%) | 199 (99%) | 208 (95%) | 192 (95%) | 199 (99%) | 164 (81%) | 183 (92%) | 174 (87%) | 196 (96%) |
| Skin rash | 179 (89%) | 182 (91%) | 193 (89%) | 190 (94%) | 176 (87%) | 189 (94%) | 185 (93%) | 167 (84%) | 167 (82%) |
| Moves only when stimulated or no movement even on stimulation | 139 (69%) | 171 (85%) | 205 (94%) | 157 (77%) | 168 (83%) | 164 (81%) | 171 (86%) | 162 (81%) | 102 (50%) |
| Not passing first stool within first 48 hours of life | 132 (66%) | 157 (78%) | 188 (86%) | 158 (78%) | 159 (79%) | 183 (91%) | 165 (83%) | 157 (79%) | 119 (58%) |
| Blood in stool | 196 (98%) | 200 (100%) | 214 (98%) | 201 (99%) | 195 (97%) | 201 (100%) | 192 (96%) | 161 (81%) | 199 (98%) |
| Diarrhea | 178 (89%) | 174 (87%) | 214 (98%) | 166 (82%) | 195 (97%) | 152 (75%) | 127 (64%) | 158 (79%) | 202 (99%) |
| Anus imperforation | 194 (97%) | 200 (100%) | 217 (100%) | 192 (95%) | 195 (97%) | 201 (100%) | 196 (98%) | 163 (82%) | 200 (98%) |
| Male newborn is not able to pass urine in a stream | 128 (64%) | 117 (58%) | 206 (94%) | 169 (83%) | 128 (63%) | 190 (94%) | 167 (84%) | 159 (80%) | 63 (31%) |
| Not able to pass urine for 24 hours | 166 (83%) | 181 (90%) | 209 (96%) | 181 (89%) | 172 (85%) | 195 (97%) | 186 (93%) | 153 (77%) | 172 (84%) |
| Dehydration signs | 194 (97%) | 190 (95%) | 214 (98%) | 190 (94%) | 183 (91%) | 199 (99%) | 196 (98%) | 167 (84%) | 204 (100%) |
| ^1^ n (%) | | | | | | | | | |

| Supplementary table 6: Comparison of Original vs Reduced Model | | | |
| --- | --- | --- | --- |
| Metric | Original Model | Reduced Model | Change |
| Model Fit |  |  |  |
| R-squared | 0.131 | 0.158 | +20.6% |
| Adjusted R-squared | 0.111 | 0.137 | +23.4% |
| Residual Std. Error | 2.379 | 1.719 | -27.7% |
| F-statistic | 6.44 | 7.59 | +17.9% |
| AIC | 8253.34 | 6717.13 | -1536.21 |
| BIC | 8489.55 | 6951.04 | -1538.51 |
| The reduced model excludes 94 influential observations (Cook's distance > 4/n = 0.0022) identified from the original model. All metrics show substantial improvement after exclusion of influential points, particularly AIC (decrease of 1,536 points) and residual standard error (decrease from 2.38 to 1.72). The 10-fold cross-validated R-squared (0.111 ± 0.050) confirms the model's generalizability, though slightly lower than the apparent R-squared (0.158), indicating modest optimism in the original fit. MAPE of 10.6% indicates that typical prediction errors are approximately 10.6% of the mean knowledge score. | | | |

**Supplementary table 7: Univariate and Multivariable Linear Regression Analysis of Factors Associated with Neonatal Danger Signs Knowledge Score (Non reduced model)**

| Variable | Level | Crude β (95% CI) | Crude p-value | Adjusted β (95% CI) | Adjusted p-value |
| --- | --- | --- | --- | --- | --- |
| Governorate (ref: New Valley) | Alexandria | 0.60 (-0.12 to 1.09) | 0.1 | 0.69 (0.11 to 1.27) | 0.020* |
|  | Asyut | 0.69 (0.21 to 1.18) | 0.005** | 0.88 (0.31 to 1.45) | 0.002** |
|  | Beheira | 1.19 (0.71 to 1.66) | <0.001*** | 0.90 (0.29 to 1.51) | 0.004** |
|  | Cairo | 0.60 (0.12 to 1.09) | 0.014* | 0.80 (0.25 to 1.34) | 0.004** |
|  | Dakahlia | 0.44 (-0.04 to 0.92) | 0.075 | 0.04 (-0.55 to 0.63) | 0.894 |
|  | Fayoum | 0.74 (0.25 to 1.22) | 0.003** | 0.87 (0.25 to 1.48) | 0.006** |
|  | Giza | 0.26 (-0.23 to 0.74) | 0.299 | 0.27 (-0.31 to 0.84) | 0.359 |
|  | Minya | -0.51 (-0.99 to -0.02) | 0.040* | -0.89 (-1.51 to -0.27) | 0.005** |
| Urbanity (ref: Urban) | Rural | 0.99 (0.23 to 1.76) | 0.011* | 0.80 (0.51 to 1.09) | <0.001*** |
|  | Remote area | 0.60 (-0.16 to 1.36) | 0.123 | -0.11 (-0.92 to 0.69) | 0.780 |
| Mother Age (ref: <20 years) | 20-24 years | -0.16 (-0.59 to 0.27) | 0.468 | -0.05 (-0.47 to 0.36) | 0.802 |
|  | 25-29 years | 0.25 (-0.17 to 0.67) | 0.246 | 0.32 (-0.11 to 0.74) | 0.143 |
|  | 30-34 years | 0.03 (-0.43 to 0.48) | 0.912 | 0.11 (-0.35 to 0.57) | 0.645 |
|  | ≥35 years | 0.47 (-0.05 to 1.00) | 0.078 | 0.67 (0.13 to 1.20) | 0.015* |
| Marital Status (ref: Single) | Married | 0.47 (-0.38 to 1.31) | 0.28 | Not included (p≥0.20) |  |
|  | Widow | 1.20 (-0.37 to 2.77) | 0.133 | Not included (p≥0.20) |  |
| Mother Education (ref: High school or lower) | University or higher | 0.24 (-0.03 to 0.50) | 0.08 | -0.19 (-0.53 to 0.16) | 0.282 |
| Father Education (ref: High school or lower) | University or higher | 0.11 (-0.15 to 0.37) | 0.403 | — | — |
| Mother Employment (ref: No) | Employed | 0.56 (0.24 to 0.87) | <0.001*** | 0.35 (0.01 to 0.70) | 0.045* |
| Father Job (ref: Causal/Unemployed) | Business owner | -0.28 (-0.64 to 0.09) | 0.138 | -0.01 (-0.40 to 0.38) | 0.946 |
|  | Employee | 0.66 (0.32 to 1.00) | <0.001*** | 0.30 (-0.08 to 0.69) | 0.117 |
|  | Worker | 0.52 (0.19 to 0.86) | 0.002** | 0.42 (0.09 to 0.74) | 0.013* |
| Work Hours (ref: <10 hours) | 10-19 hours | -0.13 (-0.39 to 0.12) | 0.303 | — | — |
|  | 20-29 hours | -0.16 (-0.78 to 0.47) | 0.625 | — | — |
|  | ≥30 hours | — | — | — | — |
| Number of Children (ref: 1 child) | 2 children | 0.20 (-0.11 to 0.51) | 0.203 | — | — |
|  | 3 children | 0.01 (-0.32 to 0.34) | 0.953 | — | — |
|  | 4 children | 0.25 (-0.18 to 0.69) | 0.251 | — | — |
|  | ≥5 children | 0.00 (-0.59 to 0.60) | 0.999 | — | — |
| Family Income (ref: Insufficient) | Insufficient & take loans | -0.17 (-0.59 to 0.26) | 0.443 | -0.22 (-0.65 to 0.21) | 0.326 |
|  | Sufficient | 0.48 (0.20 to 0.76) | <0.001*** | 0.41 (0.11 to 0.72) | 0.008** |
|  | Sufficient and save | 0.75 (0.27 to 1.23) | 0.002** | 0.37 (-0.16 to 0.90) | 0.173 |
| Baby Gender (ref: Female) | Male | 0.19 (-0.04 to 0.43) | 0.1 | 0.13 (-0.10 to 0.35) | 0.261 |
| Baby Age (ref: <6 months) | 6-11.9 months | 0.13 (-0.16 to 0.41) | 0.383 | -0.01 (-0.29 to 0.27) | 0.945 |
|  | 12-17.9 months | 0.19 (-0.13 to 0.51) | 0.239 | 0.01 (-0.31 to 0.32) | 0.956 |
|  | 18-23.9 months | 0.39 (0.01 to 0.77) | 0.046* | 0.12 (-0.27 to 0.50) | 0.553 |
|  | ≥24 months | — | — | — | — |
| Place of Delivery (ref: Government hospital) | Private hospital | 0.54 (0.28 to 0.81) | <0.001*** | 0.14 (-0.16 to 0.45) | 0.354 |
|  | Private clinic | 0.42 (0.12 to 0.73) | 0.007** | 0.55 (0.17 to 0.92) | 0.004** |
|  | Home | -0.02 (-0.95 to 0.91) | 0.966 | 0.31 (-0.61 to 1.23) | 0.513 |
| Delivery Type (ref: NVD) | CS | -0.15 (-0.44 to 0.14) | 0.306 | — | — |
| ANC Visits (ref: None) | 1-3 visits | — | — | — | — |
|  | ≥4 visits | 0.54 (0.29 to 0.80) | <0.001*** | 0.50 (0.19 to 0.80) | 0.002** |
| Father ANC Attendance (ref: All visits) | Limited visits (<4) | 0.25 (-0.07 to 0.57) | 0.119 | 0.18 (-0.14 to 0.49) | 0.272 |
|  | Did not remember/none | -0.13 (-0.45 to 0.19) | 0.428 | 0.08 (-0.28 to 0.44) | 0.664 |
| Father Pediatric Attendance (ref: All visits) | Limited visits (≤2) | — | — | — | — |
|  | Did not remember/none | -0.40 (-0.63 to -0.17) | <0.001*** | -0.30 (-0.56 to -0.03) | 0.027* |
| Education Source (ref: None) | Primary healthcare center | 0.50 (0.18 to 0.81) | 0.002** | 0.12 (-0.25 to 0.49) | 0.535 |
|  | Private clinic | 1.42 (0.83 to 2.01) | <0.001*** | 0.81 (0.17 to 1.46) | 0.014* |
|  | Public hospital | 0.47 (-0.03 to 0.98) | 0.067 | -0.03 (-0.59 to 0.52) | 0.912 |
| Preferred Information Source (ref: Social Media) | Family & Friends | 0.34 (-0.01 to 0.69) | 0.059 | 0.32 (-0.14 to 0.79) | 0.176 |
|  | Internet | 0.50 (0.01 to 0.99) | 0.046* | 0.50 (0.01 to 0.99) | 0.044* |
|  | Healthcare providers | 0.80 (0.53 to 1.07) | <0.001*** | 0.95 (0.51 to 1.40) | <0.001*** |
|  | TV & Radio | -0.33 (-1.14 to 0.48) | 0.423 | -0.07 (-0.94 to 0.80) | 0.872 |

**Model diagnostics assessment of original non reduced model**

Regression diagnostic tests revealed that the linearity assumption was satisfied, as the Residuals vs Fitted plot showed a random scatter of points around the zero line with no systematic curvature, and the lowess smooth remained close to horizontal. The Durbin-Watson test statistic was 1.508, falling within the acceptable range of 1.5–2.5, indicating no significant autocorrelation and confirming independence of observations. However, the Breusch-Pagan test was significant (p < 0.001), indicating the presence of heteroscedasticity, and the Scale-Location plot showed non-constant variance of residuals. The Shapiro-Wilk test was also significant (p < 0.001), and the Q-Q plot demonstrated substantial deviation from normality with heavy tails, indicating non-normal residuals. Despite these violations, the large sample size (N = 1,796) ensures that regression coefficients remain reliable per the Central Limit Theorem, and robust standard errors (HC3) were employed to address heteroscedasticity and non-normality. All Variance Inflation Factor (VIF) values were below 1.4 (max VIF = 1.372, mean VIF = 1.133), well under the conventional threshold of 5–10, confirming no problematic multicollinearity. Cook's distance identified 94 influential observations (5.2% of the sample) exceeding the threshold of 4/n, suggesting that several points may disproportionately influence the regression coefficients; therefore, a sensitivity analysis excluding these observations was recommended to assess the robustness of the findings.
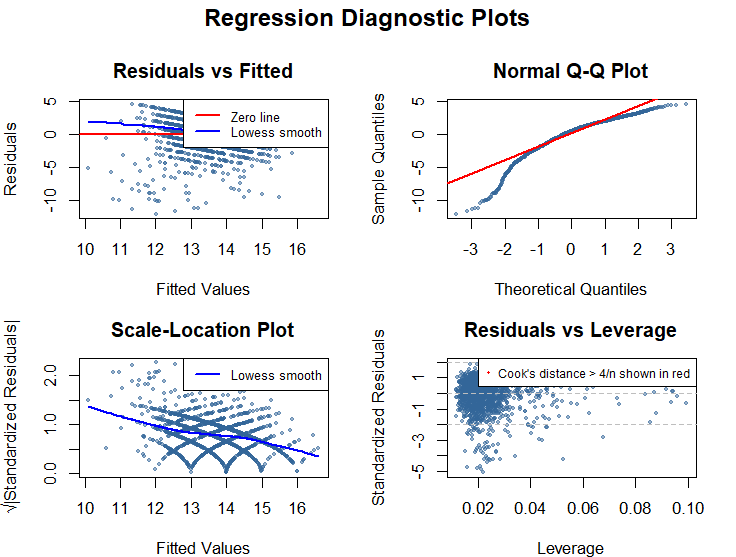


**Figure 1 supplementary: Regression Diagnostic Plots for the Reduced Multivariable Linear Regression Model (non-reduced model)**

Sensitivity analysis using Cook's distance measured the influence of each observation on the regression coefficients by quantifying the change in predicted values when that observation is excluded from the model. Observations with Cook's distance exceeding the conventional threshold of 4/n (where n is the sample size, (0.0022)) were considered potentially influential. We compared the original model (including all observations) with a reduced model excluding these influential observations. Model fit statistics, regression coefficients, and significance levels were compared between the two models to evaluate the stability of our findings. **Figure 2 supplementary**


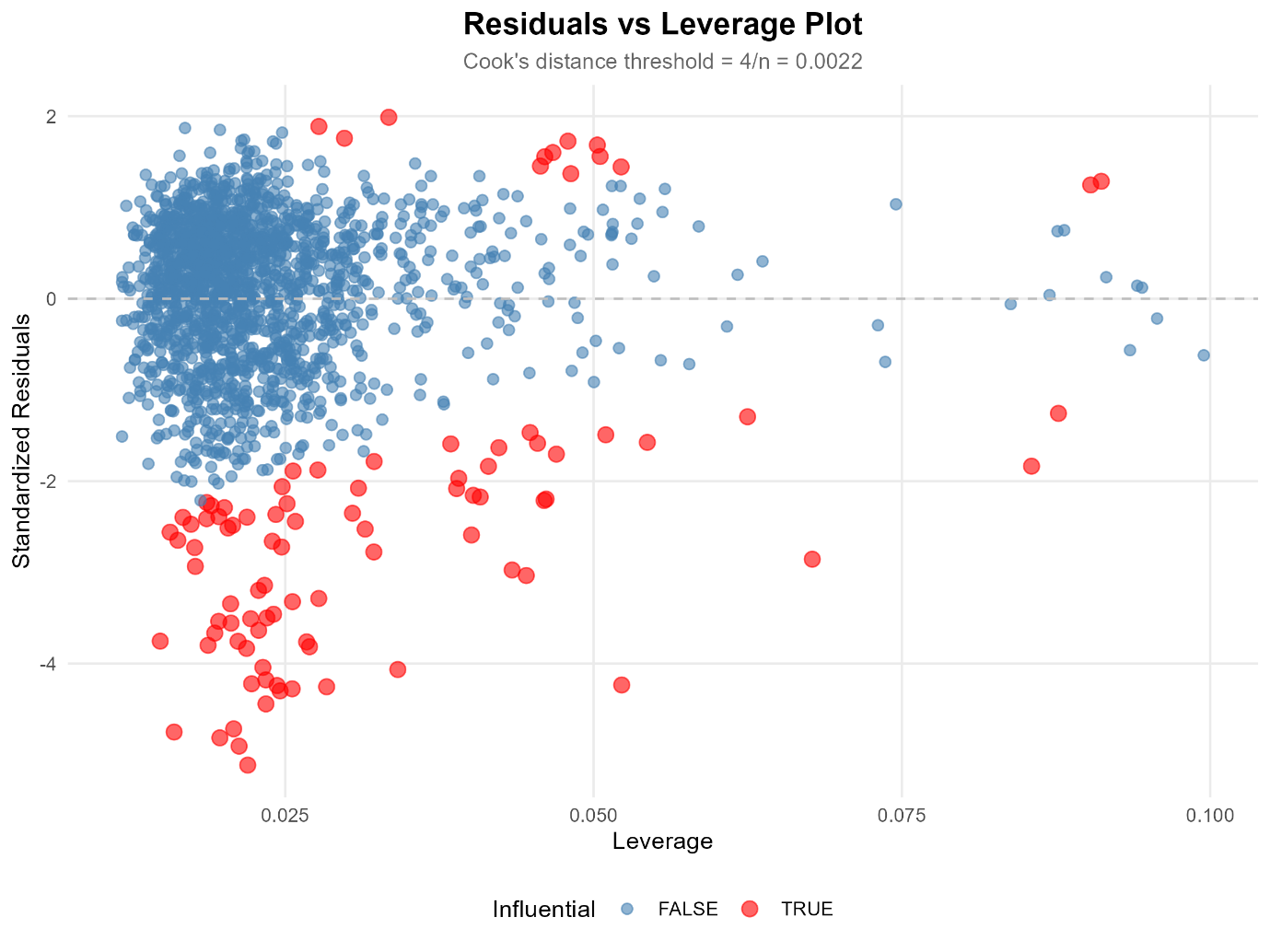


**Supplementary figure 2: Cook's Distance plot for the original multivariable linear regression model with red dots (94) representing influential observations**

A change in coefficient estimates exceeding 10% or a change in statistical significance status was considered indicative of substantial influence. A sensitivity analysis excluding observations with high influence revealed substantial instability in the model. Following exclusion of these influential points, the model fit improved markedly (R-squared increased from 0.131 to 0.158, AIC decreased from 8253 to 6717). However, 82.9% of coefficient estimates changed by more than 10%, and eight predictors changed their statistical significance status. These findings indicate that the original model results were not robust and were heavily influenced by a small subset of participants. The reduced multivariable linear regression model demonstrated statistically significant overall fit (F = 7.59, p < 0.001) and explained 15.8% of the variance in neonatal danger signs knowledge scores (R² = 0.158, adjusted R² = 0.137). The model showed good predictive accuracy with a root mean square error of 1.70 and a mean absolute percentage error of 10.6%. Ten-fold cross-validation confirmed the model's generalizability, yielding a mean cross-validated R² of 0.111 (SD = 0.050), which is only slightly lower than the apparent R², indicating minimal overfitting. Compared to the original model, the reduced model showed substantial improvements across all fit indices: R² improved by 20.6%, AIC decreased by 1,536 points, and residual standard error decreased by 27.7%. **Supplementary table 5**

**Model diagnostics assessment of the reduced model**

The Residuals vs Fitted plot shows residuals randomly scattered around the zero line with no clear systematic pattern or curvature. The lowess smooth line remains close to the horizontal zero line across all fitted values, indicating that the **linearity assumption is satisfied**. The Q-Q plot shows substantial deviation from the theoretical diagonal line, particularly at both tails. The points depart from the reference line, indicating **heavy tails** (more extreme values than expected under normality). However, given the large sample size (n = 1,702), the Central Limit Theorem ensures that regression coefficients remain unbiased, and robust standard errors were employed to address this violation. The Scale-Location plot displays a relatively flat horizontal line with a slight upward trend, indicating **non-constant variance of residuals**. This violation was addressed by using heteroscedasticity-consistent robust standard errors (HC3). "Within the reduced dataset, no observation exceeded the threshold for extreme influence (Cook's D > 0.01), and none were removed as they did not distort the model's conclusions. **Supplementary figure 3**

| 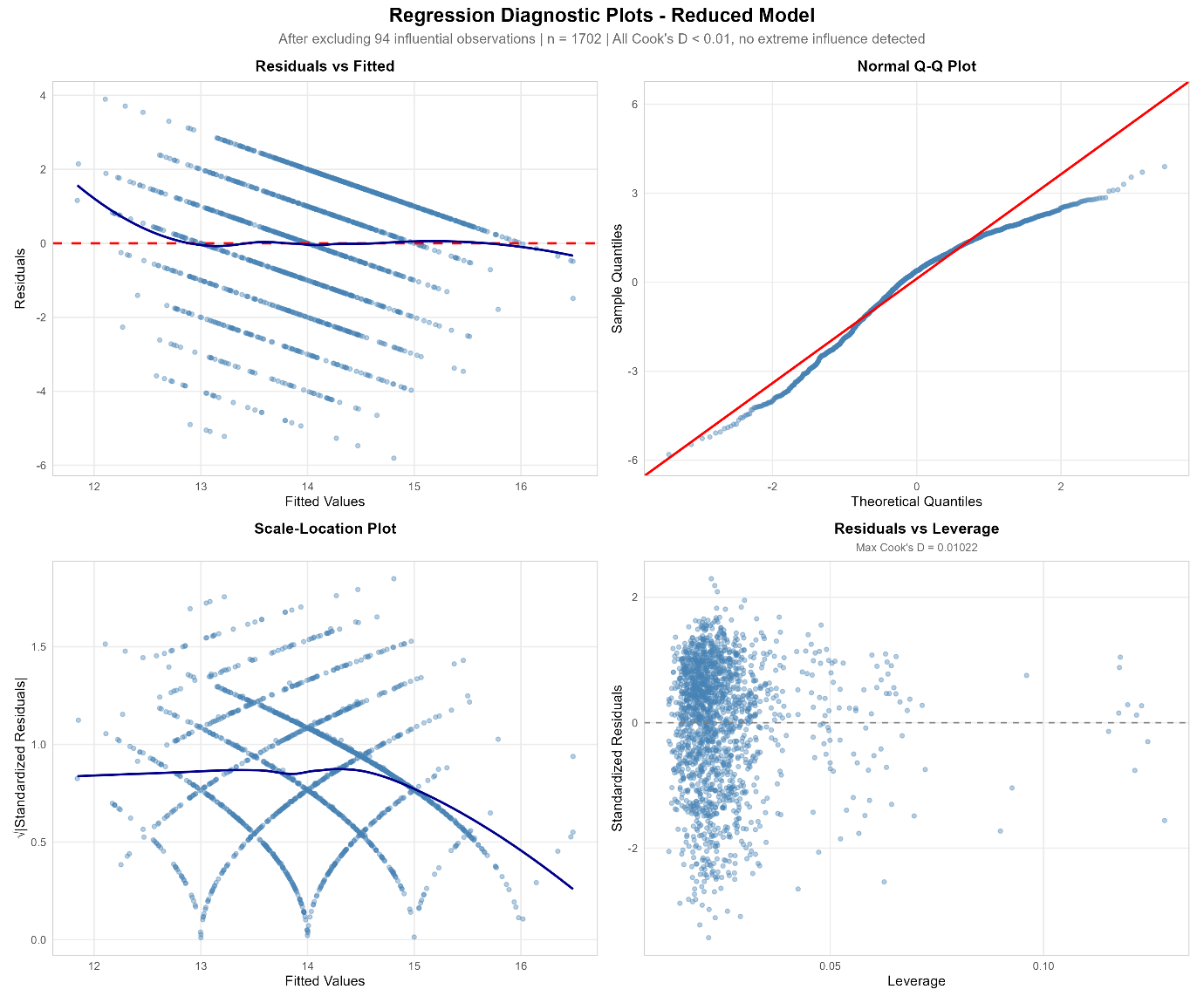 |
| --- |
| **Supplementary figure 3**: **Regression Diagnostic Plots for the Reduced Multivariable Linear Regression Model** |
